# Poloxamer 338 Affects Cell Adhesion and Biofilm Formation in *Escherichia coli*: Potential Applications in the Management of Catheter-Associated Urinary Tract Infections

**DOI:** 10.3390/pathogens9110885

**Published:** 2020-10-25

**Authors:** Mariarita Stirpe, Benedetta Brugnoli, Gianfranco Donelli, Iolanda Francolini, Claudia Vuotto

**Affiliations:** 1 Microbial Biofilm Laboratory, IRCCS Fondazione Santa Lucia, 00143 Rome, Italy; mariaritastirpe@gmail.com (M.S.); g.donelli@hsantalucia.it (G.D.); 2 Department of Chemistry, Sapienza University of Rome, 00185 Rome, Italy; benedettabrugnoli@hotmail.com

**Keywords:** poloxamer 338, biofilm, urinary catheter, *Escherichia coli*, antifouling coatings

## Abstract

Poloxamers are nontoxic, amphiphilic copolymers used in different formulations. Due to its surfactant properties, Poloxamer 338 (P388) is herein proposed as a strategy to avoid biofilm formation often causing recalcitrant catheter-associated urinary tract infections (CAUTI). The aim is to evaluate the ability of P388 coatings to affect the adhesion of Ec5FSL and Ec9FSL *Escherichia coli* strains on silicone urinary catheters. Attenuated total reflection infrared spectroscopy, atomic force microscopy, and static water contact angle measurement were employed to characterize the P388-coated silicone catheter in terms of amount of P388 layered, coating thickness, homogeneity, and hydrophilicity. In static conditions, the antifouling power of P388 was defined by comparing the *E. coli* cells adherent on a hydrophilic P388-adsorbed catheter segment with those on an uncoated one. A P388-coated catheter, having a homogeneous coverage of 35 nm in thickness, reduced of 0.83 log_10_ and 0.51 log_10_ the biofilm of Ec5FSL and Ec9FSL, respectively. In dynamic conditions, the percentage of cell adhesion on P388-adsorbed silicone channels was investigated by a microfluidic system, simulating the in vivo conditions of catheterized patients. As a result, both *E. coli* isolates were undetected. The strong and stable antifouling property against *E. coli* biofilm lead us to consider P388 as a promising anti-biofilm agent for CAUTIs control.

## 1. Introduction

Some of the most common infections acquired in both acute care hospitals and post-acute healthcare settings, such as the rehabilitation units, are the urinary tract infections (UTIs), representing around 40% of healthcare-associated infections (HAIs) [1] and being attributable for around 75% to use of an indwelling urinary catheter [2]. The most important risk factor for developing catheter-associated urinary tract infections (CAUTIs) is the prolonged use of an indwelling catheter, followed by female sex, diabetes mellitus, catheter insertion outside the operating room, and a breach in the closed system of catheter drainage [3]. In fact, it is now certain that the longer the catheter is in place, the higher the incidence of UTI. A number of diseases require long-term bladder catheterization, both those related to acute pathologies that have damaged the bladder, and those associated to chronic conditions, such as cerebrovascular accidents, spinal injury or neurological diseases, including Alzheimer, Parkinson, and Multiple Sclerosis, often causing an impaired emptying of the bladder (neurogenic bladder) and related ureteric reflux [4,5,6].

The bacterial species most frequently involved in CAUTIs are *Escherichia coli* [7], *Proteus mirabilis*, *Enterococcus spp*., *Klebsiella pneumoniae*, *coagulase-negative Staphylococcus*, *Pseudomonas aeruginosa*, *Acinetobacter baumannii*, and *Candida spp*. [8]. It is increasingly common, especially in hospitalized patients, to isolate from their urines multidrug- and extensively drug-resistant microorganisms, such as extended-spectrum beta-lactamase (ESBL)-producing bacteria, AmpC β-lactamases producers, and carbapenem-resistant Enterobacteriaceae [9,10,11].

The most important cause of CAUTI is the biofilm formation by these microorganisms along both interior and exterior catheter surfaces [12]. The establishment of biofilm communities on urinary catheters is considered the cause of chronic and recurrent UTIs, since they are able to bypass host defense mechanisms and tolerate antibiotic/antifungal therapies [13]. The resulting underestimated antimicrobial tolerance and the misuse of antibiotics trigger a vicious circle in which microorganisms acquire an even greater drug resistance.

No commercially available antibiotic has been demonstrated to be fully effective against bacteria grown as biofilm on urinary catheters. To overcome that, in the past few decades a great effort has been devoted to the development of safe, effective, and fast-acting anti-biofilm molecules and strategies that do not confer evolutionary pressure to microorganisms [14] as well as antifouling and antimicrobial coatings for medical devices, such as urinary catheters, for the purpose of improving their long-term and broad-spectrum efficacy, and biocompatibility [15].

Poloxamers are inert amphiphilic tri-block copolymers [poly(ethylene oxide)-block-poly(propylene oxide)-block-poly(propylene oxide) (PEO-PPO-PEO)] reported as suitable compounds for wound dressing, with a number of papers exploring the use and mode of action of mostly Poloxamer 188 (Pluronic F-68) and Poloxamer 407 (Pluronic F-127) in the enhancement of wound healing and their effect on biofilms [16]. Poloxamer ability to promote wound healing has been attributed to several factors including wound cleansing, suppressing protein aggregation, repairing tissue or cell membranes, and exerting intrinsic antimicrobial activity [17]. Poloxamer 407 was also employed to stabilize silver nanoparticles and develop formulations for skin wounds’ care [18].

Nevertheless, it has been demonstrated that when applied to hydrophobic surfaces, such as silicone, poloxamers can self-assemble at the water/surface interface in a brush-like configuration, where the hydrophobic polypropylene oxide (PPO) block anchors to the surface while the hydrophilic polyethylene oxide (PEO) chains dangle in the adjacent solution, drastically reducing the nonspecific proteins adsorption [19] and bacterial adhesion [20]. Indeed, the hydrophilic PEO is considered the gold standard for antifouling applications, its repelling activity being related to hydration and steric hindrance effects [21]. 

In this study, a different type of poloxamer, Poloxamer 338 (P388), was chosen as potential antifouling coating for urinary catheters, because of its high PEO content (80%) that results in an HLB (hydrophilic–lipophilic balance) higher than that of the commonly employed surfactant Pluronic F127 [22]. Specifically, P388 was adsorbed onto both silicone urinary catheters and silicone tubes comparable to urinary catheters, in order to evaluate the anti-biofilm efficacy against *E. coli* strains in static and dynamic conditions, respectively. 

## 2. Results

**Figure 1:**
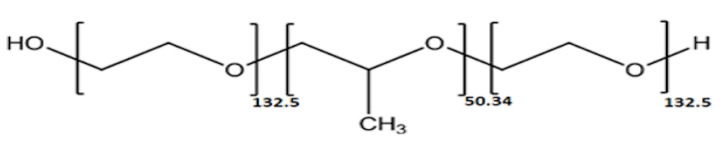
Chemical structure of Poloxamer 388 (P388). The P388 is a nontoxic, amphiphilic triblock copolymer composed of a central hydrophobic chain of poly (propylene oxide) flanked by two hydrophilic chains of poly (ethylene oxide).

In this study, the efficacy of the P388 poloxamer (Figure 1) as antifouling coating for silicone urinary catheters was investigated against biofilm-forming *E. coli* strains causing CAUTIs. Among the different available poloxamers, differing in terms of PPO units (ranging from 16 to 69) and molecular weight (ranging from 1850 to 14,600), P388 (also known as F108) was chosen because it possesses a significantly high number of PPO units (ca. 50) and the highest molecular weight (14,600). 

The potential antifouling ability of P388 was investigated in both static and flow conditions. In particular, the static conditions in an open model allowed us to fully characterize the chemical properties of the P388-modified silicone surfaces, while the flow conditions applied in the Bioflux system permitted to study biofilm formation under a shear rate that is a typical condition in urinary catheterization. 

**Figure 2:**
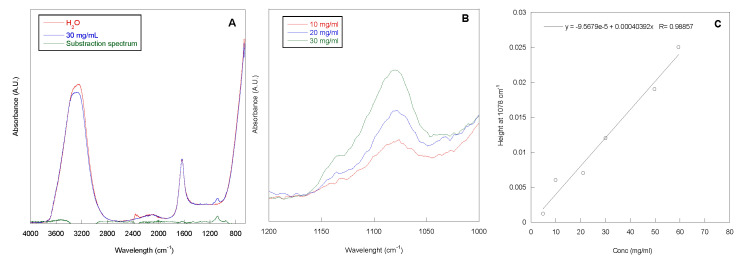
ATR-IR spectra. ATR-IR spectra of water, P388 water solution at 30 mg/mL and the relative subtraction spectrum (**A**). ATR-IR spectra of P388 water solution at different concentrations in the 1200–1000 cm^−^^1^ spectral range (**B**). Calibration curve peak height at 1078 cm^−^^1^ vs. P388 concentration (**C**).

The adsorption grade of P388, at concentrations ranging from 5 to 60 mg/mL, onto 1 cm long segment of silicone catheter was determined by Attenuated Total Reflection Infrared Spectroscopy (ATR-IR) analysis of the P388 solutions before and after contact with catheter. In Figure 2A, the ATR-IR spectrum of the P388 solution at 30 mg/mL is reported. In the spectrum, the adsorption peaks of water (stretching at ca. 3200 cm^−^^1^ and bending at ca. 1600 cm^−^^1^) and a peak centered at 1078 cm^−^^1^, related to the C–O–C stretching of PEO and PPO units, are present. This latter peak is more evident in the subtraction spectrum reported in Figure 2A, obtained by subtracting the spectrum of H_2_O to the spectrum of the P388 30 mg/mL solution. In Figure 2B, the spectra of three selected P388 solution concentrations (10, 20, and 30 mg/mL) in the 1200–1000 cm−1 spectral range are reported. 

As it can be observed, the intensity of the peak at 1078 cm^−^^1^ increased with P388 solution concentration. This is in accordance with the Lambert–Beer equation (*A =* *ε x c x l*; where *A* is the absorbance at the chosen wavelength, *c* is concentration of the sample, *ε* the molar extinction coefficient, and *l* is the optical length that in our case corresponds to the beam penetration depth). Therefore, a calibration curve height of the peak at 1078 cm^−^^1^ vs. concentration was obtained with a good correlation coefficient (R) (Figure 2C). Such a curve was used to determine the unknown P388 concentration after contact with the segment of silicone catheter to be subtracted from the solution concentration before catheter contact. 

**Figure 3:**
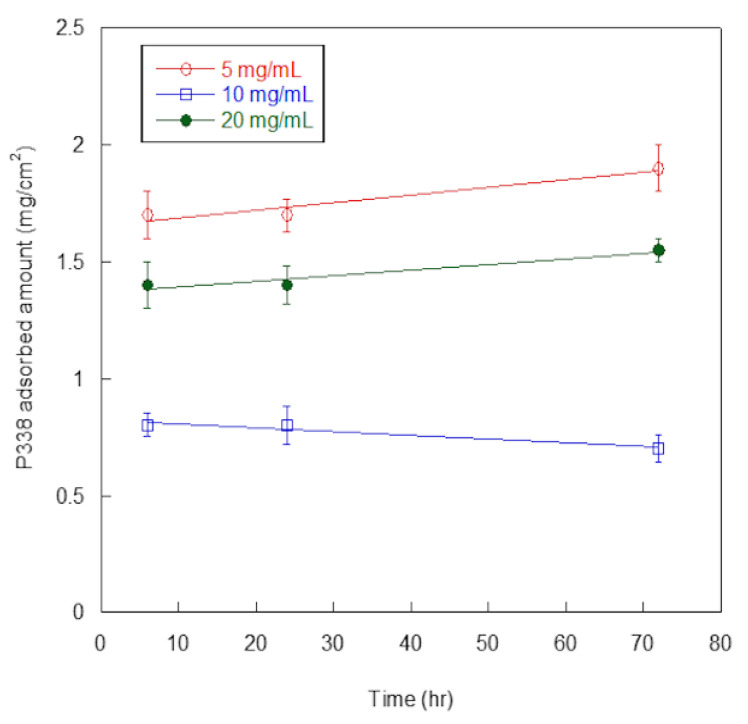
P388 adsorption on silicone. Quantification of P388 adsorbed per surface unit (cm^2^) of silicone catheter. Results are expressed as means ± standard deviations of three independent experiments.

In Figure 3, P388-adsorbed amounts, expressed as mg per surface unit (cm^2^), are reported as a function of P388 concentration and time of adsorption.

At all of the explored concentrations (5, 10, and 20 mg/mL), adsorbed P388 amount was similar at 6 h and 24 h, and increased only slightly at 72 h, except for 10 mg/mL concentration. Such finding suggested the adsorption was fast so that the maximum adsorption was already reached at 6 h. Additionally, at any time of adsorption (6, 24, or 72 h), the highest adsorption amounts were obtained at 5 mg/mL P388 concentration. Presumably, the desorption process competed with the adsorption one at higher P388 concentrations. 

For these reasons, we decided to use 5 mg/mL (0.5%) as working concentration of P388 and 6 h as adsorption time for further characterization and experiments. In these conditions, the P388-adsorbed amount was 1.7 ± 0.2 mg/cm^2^, that is 18.7% of the P388 amount available for adsorption.

**Figure 4:**
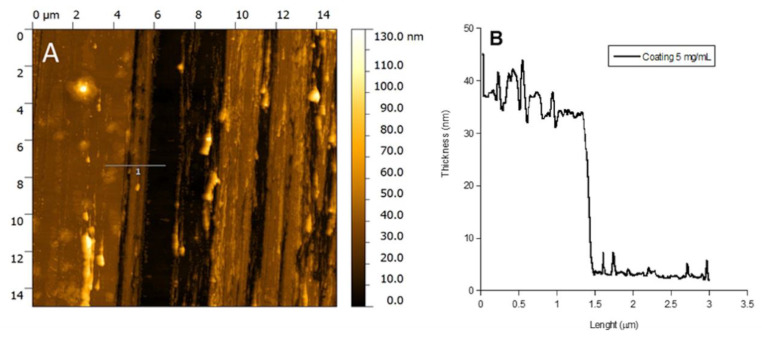
AFM analysis. AFM image of P388 coating obtained at 5 mg/mL P388 concentration (**A**) and coating height profile at the same concentration (**B**).

In Figure 4, the Atomic Force Microscopy (AFM) image and the thickness profile of the P388 catheter coating obtained at 5 mg/mL P388 concentration are reported. The black path present in the image is the one done by scratching the surface with a needle. As it can be observed, the coating is homogeneous and with a thickness of ca. 35 nm (Figure 4B). 

The contact angle of the uncoated and coated catheter surfaces was also evaluated in order to gain information about surface wettability before and after coating. Indeed, surface hydrophilicity is known to strongly affect bacterial adhesion and biofilm formation. The uncoated catheter had a contact angle (θ°) of 95° ± 5, confirming the hydrophobicity of the silicone surface, while catheters exposed to P388 solutions at 5 mg/mL for 6 h showed a contact angle of 72° ± 3, suggesting the exposure of the PEO hydrophilic chains at the water/surface interface. It is then possible to speculate that the adsorbed P388 was in a brush-like configuration promoted by the high hydrophobicity of silicone [23]. The brush-like configuration is the preferred one for our purposes since the hydrophilic PEO brushes could hinder bacterial adhesion by hydration and steric hindrance effects [24].

The two *E. coli* isolates (Ec5FSL and Ec9FSL) to be tested were selected on the basis of the antibiotic resistance profile and the biofilm-forming ability. 

Looking at the antibiotic resistance profile, the Ec5FSL isolate was classified as susceptible (S), being susceptible to all the tested classes of antibiotics; while Ec9FSL was considered multidrug-resistant (MDR), since the isolate was nonsusceptible to at least one agent in ≥3 antimicrobial categories (fluoroquinolones, penicillins, third-generation cephalosporins, fourth-generation cephalosporin). 

**Figure 5:**
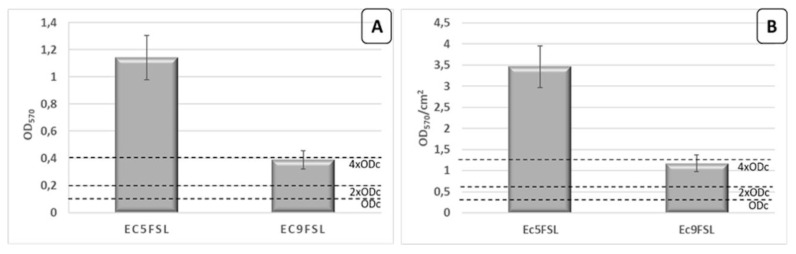
Biofilm production assay. Quantification of Ec5FSL and Ec9FSL biofilms formed after 20 h incubation on 96-well plates, expressed as OD_570_ (**A**) and taking into consideration the cell growth area of each well (0.33 cm^2^) (**B**). Results are expressed as means ± standard deviations of three independent experiments.

Regarding the phenotypic characterization, Ec5FSL isolate was classified as strongly adherent on polystyrene 96-well plate while Ec9FSL isolate as moderately adherent (Figure 5A). The results were also expressed as Optical Density (OD)_570_ per surface unit (cm^2^) (Figure 5B). 

The Ec5FSL susceptible isolate was more able to grow as biofilm with respect to the MDR one, this possibly being a compensation method to enhance its long-term survival within the host.

**Figure 6:**
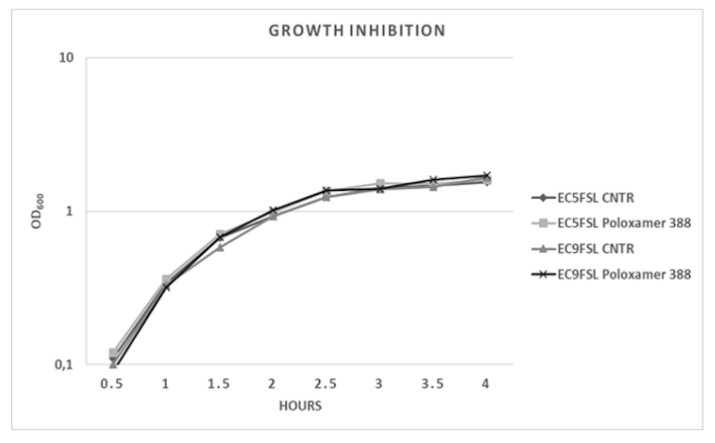
*E. coli* growth kinetics against P388. Growth curves of Ec5FSL and Ec9FSL alone and in presence of P388. Absorbance unit is on a logarithmic scale.

To determine a possible growth inhibition activity of this molecule, the two isolates were grown in presence of P388 solution at 5 mg/mL concentration (Figure 6). P388 did not have significant intrinsic activity against the two tested strains (Figure 6).

**Figure 7:**
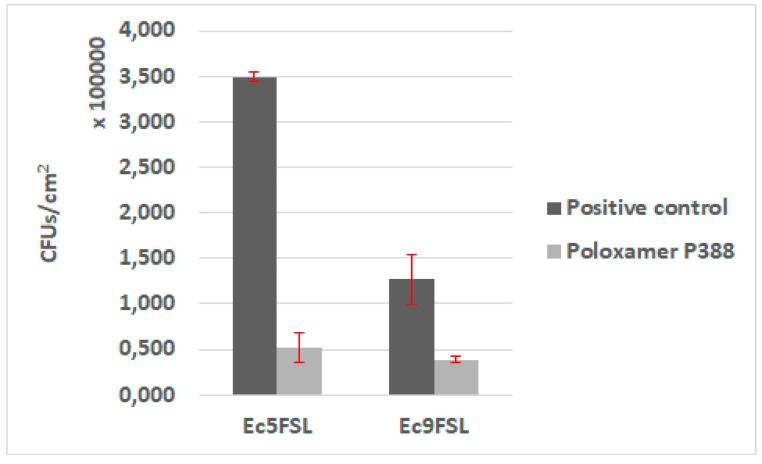
Antifouling activity of P388 in static conditions. CFU/cm^2^ results of Ec5FSL and Ec9FSL biofilms formed on P388-adsorbed segment of silicone urinary catheter (Poloxamer 388) and on unadsorbed catheter (positive control).

The antifouling activity of P388 was first assessed in static conditions, by adsorbing the molecule on a 1 cm segment of 100% silicone catheter. Biofilms of Ec5FSL and Ec9FSL grown on pristine silicone catheter and P388-adsorbed silicone catheter were collected to determine the bacterial CFU/cm^2^ on silicone catheter (Figure 7). 

A statistically significant reduction of the cells adhered on P388-adsorbed catheter was detected in both isolates. The stronger evidence was observed in Ec5FSL with a *p*-value of 0.0003, while the reduction of Ec9FSL biofilm was *p*-value of 0.0022. Regardless, a 99% level of statistical significance (*p*-value ≤0.01) was determined for both of them. More specifically, about an 85% (0.83 log_10_) and 78% (0.65 log_10_) reduction in CFU/cm^2^ of Ec5FSL and Ec9FSL, respectively, compared with the untreated controls (100%), was observed when P388 was absorbed onto silicone catheters (Figure 7). 

**Figure 8:**
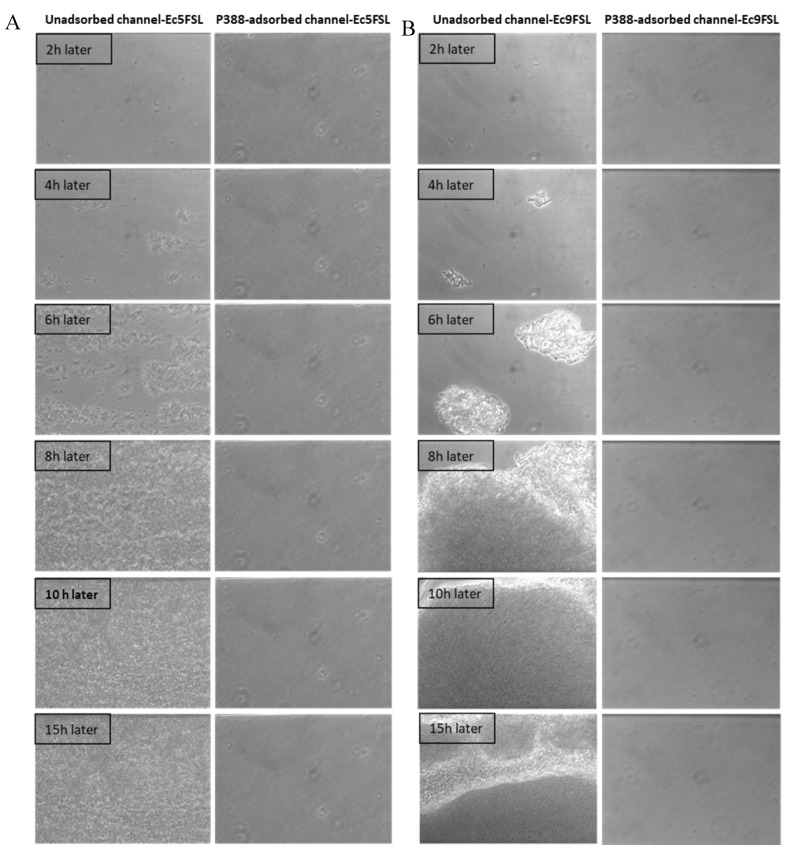
Antifouling activity of P388 in dynamic conditions. Bioflux micrographs taken at 20× objective magnification, at different time points, of Ec5FSL (**A**) and Ec9FSL (**B**) biofilm formation on unadsorbed (left side of semicolumn) or P388-adsorbed (right side of semicolumn) silicone channels after 15 h under a shear flow of 0.5 dyn/cm^2^ (0.05 Pa).

The efficacy of P388 in reducing the *E. coli* adhesion on a silicone-made urinary catheter was further assessed by using a microfluidic apparatus (BioFlux System 200, Fluxion Biosciences, Inc., Alameda, CA, USA) that allows a dynamic biofilm analysis under controllable shear forces. To investigate the potential ability to interfere with the initial microbial adhesion process, P388 was adsorbed onto the silicone microfluidic flow channels arranged on a BioFlux well plate. In this way, the in vivo conditions of urinary catheters were mimicked in terms of both flow conditions and catheter material. As reported in the Materials and Methods section, P388 adsorption onto the silicone microfluidic flow channels was carried out by using a 5 mg/mL P388 concentration, that is, the concentration which gave the best results in static experiments. However, it should be considered that the maximum P388 solution volume able to fill the flow channel was significantly lower with respect to the volume used in static experiments (few microliters instead of 3 mL), this meaning that, at the same starting concentration, the P388 amount (mg) available for adsorption to flow channel surface unit (cm^2^) was only 0.03 mg/cm^2^ compared with 9.1 mg/cm^2^ of static experiments. The decision to keep 5 mg/mL P388 concentration arises from the fact that it would not have been possible in any case to reach in dynamic conditions a P388 availability of 9.1 mg/cm^2^ without exceeding the P388 critical micelle concentration. Therefore, even if not experimentally definable because of the closed system used which makes direct measurement impossible, we expect on the microfluidic flow channels a P388 coating significantly less concentrated than that obtained on the catheter segment. As shown in Figure 8, the P388 coating, although at very low concentrations, was able to protect the silicone surface from cell adhesion and biofilm formation by both Ec5FSL and Ec9FSL isolates. Indeed, as shown in Figure 8, biofilms of both strains achieved full surface coverage of uncoated silicone channels within 15 h, while the cells adhesion forces were definitely low on P388-adsorbed silicone microfluidic tubes, they were enough to completely inhibit biofilm formation under shear forces.

**Figure 9:**
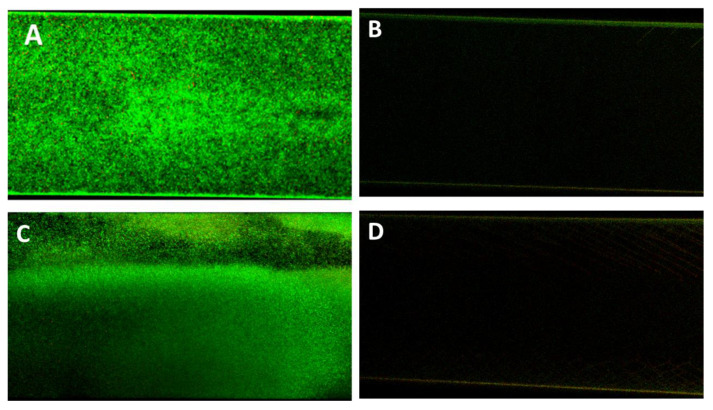
Antifouling activity of P388 in dynamic conditions. Representative CLSM images of Ec5FSL (**A**,**B**) and Ec9FSL (**C**,**D**) biofilms formed after 15 h under a shear flow of 0.5 dyn/cm^2^ (0.05 Pa) on unadsorbed (**A**,**C**) and P388-adsorbed (**B**,**D**) microfluidic channel.

In confirmation of the Bioflux micrographs, Figure 9 shows the Confocal Laser Scanning Microscopy (CLSM) images of the P338-adsorbed and unadsorbed channels obtained after live/dead staining. As can be seen, the only detectable biofilm was that formed within the unadsorbed channel (Figure 8A,B), whilst in P388-adsorbed channel it was only detectable a negligible amount of live/dead stain adsorbed by the silicone tube walls.

As it can be inferred from the Table 1, as predictable, the area fraction covered by biofilm was nil for both Ec5FSL and Ec9FSL isolates with respect to their controls. 

In addition, the area fraction was also nil when tests were carried out after a sterile LB washing step for 14 h under a shear flow of 0.5 dyn/cm^2^ within P388-adsorbed channel (data not shown since results were similar to Figure 8), this proving also the stability of the P388 adsorption on silicone surfaces related to the strong hydrophobic interactions between silicone and the PPO segment of P388.

P388 coating resulted to be also stable when artificial urine was used as flow and growing medium, by letting urine flow for 24 h inside the P338-adsorbed channel and then proceeding to inoculate the bacteria in the same medium (results comparable to Figure 8). 

By analyzing the micrographs of the first adhesion steps for both isolates in the two conditions, it was possible to observe that the number of Ec5FSL and Ec9FSL cells able to adhere to the P388-coated surface within one hour was much lower than that of cells attached on the pristine silicone surface, and that once the flow was activated the cells were unable to remain adherent, being very loosely bound.

Interestingly, as side data, the Ec9FSL isolate showed a significant phenotypic variation and an increased ability to grow as biofilm in dynamic conditions (Figure 8) with respect to static ones (Figure 5), so reaching the Ec5FSL biofilm-forming ability. This finding can be explained with an induction of biofilm upon environmental stress, the fluid shear force possibly being an environmental signal for Ec9FSL biofilm formation. Thus, it can be hypothesized an important role of the catheter microenvironment, especially the fluid flow, in the biofilm establishment by some strains of MDR *E. coli* that are not intrinsically strong biofilm producers.

## 3. Discussion

Nonionic, amphiphilic, tri-block copolymers, because of their amphiphilic structures, are known to be able to self-assemble in water at variable temperatures depending on polymer molecular weight and hydrophilic/hydrophobic molar ratio. The surfactant properties of poloxamers justify their large use as excipients in cosmetics and pharmaceuticals, where their use is considered safe with a rapid clearance from the body also when introduced via routes other than dermal exposure [25,26]. In particular, no maximum concentration of P338 use has been suggested in marketed mouthwashes and breath fresheners. Also, acute, short-term, sub chronic, and chronic animal testing suggested a low order of toxicity and little concern about carcinogenesis, with its use at 0.2 or 1.0 g/kg day producing no adverse effects in dogs [26].

The use of poloxamers as antifouling compounds for indwelling medical devices is still little evaluated. In general, just a few studies have investigated the antiadhesive properties of nonchemically derivatized Poloxamer 188 and Poloxamer 407 onto poly methyl methacrylate (PMMA) [27], polystyrene (PE) [28,29], and silicone sheets [30,31]. A study also addressed the colonization of a Poloxamer 407-coated silicone rubber discs in an infected pocket in vivo, mimicking revision surgery after biomaterial-associated infection [32].

The search for nonbiocidal compounds able to counteract biofilm formation on indwelling medical devices, causing neither toxicity nor development of resistance, is increasingly being reported in the literature. In this study was demonstrated the nonbiocidal activity of Poloxamer 388, these results being comparable with those obtained by Veyries and co-workers [27] with Poloxamer 407 against *staphylococci*. On the contrary, *Mycobacterium avium* complex (MAC), having an outer glycolipid layer which protects the organisms from antibiotics and host defense mechanisms, was inhibited by Poloxamer 331, which retarded the growth of most isolates, presumably by disrupting the lipid barrier [33]. Thus, a species- and poloxamer-specific effect can be hypothesized. On the contrary, P388 was an effective antifouling agent for inhibiting biofilm growth of *E. coli* on hydrophobic surfaces even at much lower concentrations than those necessary by using other poloxamers, such as 20% Poloxamer-188 [31].

More specifically, a P388-coated silicone urinary catheter, well characterized in terms of amount of adsorbed P388, surface properties, layer thickness, surface coverage, and coating stability, was able to significantly reduce biofilm formation of both susceptible and multi-drug-resistant *E. coli* isolates in static conditions. This result confirms the well-known antifouling properties of polyethylene oxide, a hydrophilic macromolecule resistant to nonspecific adsorption of proteins and cells. The antifouling mechanism of PEO seems to be multifactorial and related to combined hydration and steric hindrance effects [34]. Specifically, due to its high hydrophilicity, PEO adsorption or binding to surfaces reduces the interfacial free energy, thus decreasing the driving force for nonspecific biofouling. Additionally, in aqueous environment, the hydration of PEO chains results in the formation of a water layer strongly bonded to the surface, which represent a barrier for proteins and bacteria approaching the surface. As for the steric hindrance effect, Mu and colleagues demonstrated by molecular modeling that onto PEGylated surfaces PEO chain remains highly flexible and can form a distinctive hydrated polymer layer [35]. The adsorption of proteins or bacteria onto such a layer would involve the compression of PEO chains, which is a not thermodynamically favorable process. This was further confirmed by measuring the interaction forces between bacteria and PEO-coated glass surface [35]. Specifically, it has been demonstrated the absence of long-range attractive forces and the presence of repulsive forces related to steric effects [36].

The experiments carried out under flow aimed at verifying the P388 antifouling properties in conditions better resembling those of urinary catheterization. We showed that P388-coated silicone surfaces were also strongly refractory to biofilm formation under flow. P388 coating resulted to be stable, confirming the strong hydrophobic interactions established between the hydrophobic silicone surface and the PPO segments of P388. As the only term of comparison in the literature, Nejadnik and colleagues [20] evaluated the efficacy, in dynamic conditions, of another poloxamer, Pluronic F-127, against *Staphilococcus* and *Pseudomonas* biofilms formed on silicone rubber sheets, and they observed only a delay in biofilm formation on brush-coatings with respect to pristine silicone rubber surface. However, it should be noted that, in addition to the poloxamers being different, the two systems are also not completely comparable because of the silicone rubber sheets allocated in chambers used by the authors instead of the silicone tube (with a flow inside) used in this study. Additionally, concentration, timing and shear flow were different, as they exposed the silicone rubber sheet to a solution of 0.05% of Pluronic F-127 for 20 min in the adsorption step and the experiment was carried out at 0.002 Pa [20].

The coating stability was maintained in artificial urine, its use as flow medium not affecting the antifouling efficacy of P388 coating. These results imply that PEO brushes over the surface avoided the formation of a conditioning film potentially able to reduce the efficacy. Without any doubt, the most satisfactory result was obtained under dynamic conditions, when a 100% efficacy was detected, although at even lower P388-adhered concentrations than those used for static experiments. The Bioflux allowed us to obtain totally reproducible and comparable results by using hydrodynamic conditions that mimic, at a microscale, phenomena occurring in the urinary system, combined with the use of plates having microfluidic channels made of the same material as the catheters. 

## 4. Materials and Methods 

### 4.1. Materials and Bacterial Strains

P388 (Merck), also named Pluronic F108, has a molecular weight (Mn) of 14,600 Da with a number of PPO unit of 50.34 and PEO unit of 265.45 [22]. 

*E. coli* Ec5FSL and Ec9FSL strains were collected from urines of catheterized patients suffering from CAUTI hospitalized at IRCCS Fondazione Santa Lucia (Rome, Italy), and identified by VITEK^®^ 2 (bioMérieux Italia). VITEK^®^ 2 system was used for the antimicrobial susceptibility testing (AST) of Ec5FSL and Ec9FSL isolates, by performing automated Minimum Inhibitory Concentration (MIC) testing with Vitek2 card AST-N204, intended for use in the determination of antibiotic sensitivity of clinically relevant aerobic Gram-negative bacilli. 

### 4.2. Quantitative Biofilm Production Assay

Ec5FSL and Ec9FSL isolates were grown overnight at 37 °C in Luria-Bertani (LB) broth. Quantitative biofilm production assay was performed as described in Vuotto et al. [11]. Each plate was incubated for 20 h at 37 °C, and experiments were repeated three times. 

### 4.3. P388 Adsorption onto Silicone Tubes 

P388 was adsorbed onto cut lengthwise 1 cm long segments of a two-way Foley catheter (18 Fr) marketed by Unomedical (ConvaTec Company, Deeside, UK) by immersing each of them into 3 mL of P388 water solution at 5, 10, or 20 mg/mL concentrations (3.4 × 10^−^^4^ M, 6.8 × 10^−^^4^ M, and 1.4 × 10^−^^4^ M, respectively) for 6, 24, and 72 h. By considering the catheter segment size, the P388 amount available for adsorption to catheter surface unit at each P388 concentration was 9.1 mg/cm^2^, 18.2 mg/cm^2^, and 36.4 mg/cm^2^ for 5, 10, and 20 mg/mL, respectively. Following immersion, catheter segments were recovered and washed twice with PBS to remove the unadsorbed polymer. All the selected P388 concentrations were lower than polymer critical micelle concentration, which is 33.4 g/L at 25 °C [37].

### 4.4. Determination of the Amount of Adsorbed P388

The amount of adsorbed P388 was obtained by subtracting from the initial P388 concentration both the P388 amount still present in solution after the contact with cut lengthwise 1 cm segment of urinary catheter and the amount further detected in the washing solutions. To this aim, a novel analytical method based on ATR-IR (Nicolet 6700 spectrophotometer) technique was set up to determine P388 amount in solution. Specifically, a drop of P388 solution at concentration ranging from 5 to 60 mg/mL was poured over the surface of the ATR crystal, and ATR spectra were recorded in the 4000–650 nm wavelength range, at 2 cm^−^^1^ resolution and 200 accumulations. By assuming a constant refractive index for the different Poloxamer 388 concentrations, a calibration curve peak height at 1078 cm^−^^1^ vs. P388 concentration was obtained and used to determine unknown P388 amounts in solution.

### 4.5. Evaluation of Morphology and Thickness of P388 Coatings 

The morphology and thickness of P388 coatings were evaluated by AFM. Specifically, for thickness evaluation, a scratch was done onto the surface of P388-coated samples by a needle in order to create a channel and measure thickness of the polymeric layer. AFM characterization was performed by using a Veeco AFM Multimode™ equipped with a Nanoscope IIIa controller. Images were obtained in tapping mode acquiring topography, amplitude and phase data, by using a RTESP Bruker tip (nominal parameters r = 8 mm, f = 300 kHz, k = 40 N/m) and with a 512 × 512 pixels resolution. Images were then corrected by polynomial background filters using the software Gwyddion 2.31.

### 4.6. Static Contact Angle

To examine catheter wettability before and after P388 coating, the static water contact angle was measured at room temperature by the drop method. Specifically, a water droplet (Milli-Q water) was deposited on the catheter surface, and a picture was taken. Since the catheter has a curved surface, image processing, spline fitting, and numerical integration were used to extract the drop contour in a number of cross-sections so to determine the drop contact angle at the equilibrium, according to the method developed by Guilizzoni [38]. The obtained images were elaborated with the SigmaPlot software (Systat Software Inc., San Jose, CA, USA). The reported contact angle was the mean value of five measurements.

### 4.7. E. coli Growth Inhibition Assay in Presence of P388

Overnight inocula of *E. coli* Ec5FSL and Ec9FSL strains in LB broth were adjusted to OD_600_ values of 0.02 in 4 mL of sterile LB broth, and 1 mL of P388 solution at 25 mg/mL concentration was added to each culture (5 mg/mL final concentration). The ODs cultures were measured at half-hourly intervals through a 5 h incubation period under shaking at 280 RPM. Growth medium without bacteria was used as negative control, while positive controls were *E. coli* isolates grown in LB and sterile water in order to obtain equal volumes for all samples. The periodic measures were plotted to obtain a growth curve, so to determine the growth rate and generation time of Ec5FSL and Ec9FSL in presence of P388. 

### 4.8. Adherence Assay of E. coli on P388-Adsorbed Urinary Catheter in Static Conditions

The biofilm-forming ability of Ec5FSL and Ec9FSL strains on 1 cm long segments of urinary catheter adsorbed with P388 was evaluated. Each P388-adsorbed segment was placed onto the bottom of a well of a 24-well culture plate and filled in with 250 µL of strain culture, grown overnight in LB broth, adjusted to OD_600_ 0.1, and 2.25 mL of LB supplemented with glucose 1% (*w*/*v*). An unadsorbed segment inoculated with each strain at the same conditions was used as positive control, while an adsorbed segment with sterile LB broth was used as negative control. Thus, the plate was incubated for 20 h, and the biofilms grown on the segments were quantified by sonicating them in 5 mL of Ringer’s solution, vortexing for 30 sec and plating 100 µL of serial dilutions 1:10 on Muller Hinton agar. After 18 h at 37 °C, colonies were enumerated to calculate average CFU/mL. The internal and external surfaces of the 1 cm long silicone catheter segment were considered for CFU/cm^2^ calculation. The assay was performed in triplicate.

### 4.9. Adherence Assay of E. coli on P388-Adsorbed Urinary Catheter in Dynamic Conditions

P388 solution at 5 mg/mL concentration was made to flow inside the microfluidic channel of Bioflux (Fluxion Biosciences), and the flow was stopped to let it adsorb for 6 h, so to obtain a P388-adsorbed microfluidic channel. As positive control, sterile water was instilled within the channel and left for 6 h. After that, Ec5FSL and Ec9FSL strains was inoculated at OD_600_ 0.25 within the channels and left to adhere for 1 h. Nonadherent cells were pushed out of the channel towards the waste well, and the experiments were run for 15 h under a shear flow of 0.5 dyn/cm^2^ (0.05 Pa), determined on the basis of the diameter of microfluidic channel likely to be within the physiological range of hydrodynamic conditions occurring in the human urinary tract [39]. A time-lapse recording monitored the biofilm development, by taking a sequence of frames at set intervals (2 min) in order to record changes that take place slowly over time, from the beginning to the end of dynamic experiments.

### 4.10. Evaluation of the Stability of the P388 Adsorption on Silicone Surfaces after Urine Flow 

P388 solution at 5 mg/mL concentration was made to flow inside the microfluidic channel of Bioflux, and the flow was stopped to let it to adsorb for 6 h, so to obtain a P388-adsorbed microfluidic channel. After that, artificial urine was made to flow within the P388-adsorbed channel for 24 h under a shear flow of 0.5 dyn/cm^2^. Ec5FSL was inoculated at OD_600_ 0.25 within the channel and left to adhere for 1 h. Nonadherent cells were pushed out of the channel towards the waste well, and the experiments were run for 15 h under a shear flow of 0.5 dyn/cm^2^ (0.05 Pa). A time-lapse recording monitored the biofilm development, by taking a sequence of frames at set intervals (2 min) in order to record changes that take place slowly over time, from the beginning to the end of dynamic experiments.

### 4.11. Confocal Laser Scanning Electron Microscopy

*E.coli* biofilms grown within microfluidic channels were stained with LIVE/DEAD BacLight Bacterial Viability Kit (Molecular Probes) that utilizes a mixture of SYTO^®^ 9 (green-fluorescent nucleic acid stain) and propidium iodide (red-fluorescent nucleic acid stain). This kit allowed us to discriminate between live and dead cells, with intact cells staining fluorescent green, whereas damaged bacteria stained fluorescent red. In detail, a 0.85% NaCl solution with 6 μM SYTO 9 and 30 μM propidium iodide was instilled within the microfluidic channel for 30 min at 0.4 dyne/cm 2. After the staining, the channel was washed with a 0.85% NaCl solution for 20 min at 0.4 dyne/cm 2. The entire length of channel designed for Bioflux visualization, portioned in different sections, having each one an area of 0.234 mm 2, has been observed by CLSM (Nikon mod. C1si) at 20× magnification.

### 4.12. Data Analysis

For quantitative biofilm formation assay, the cut-off OD (ODc) was defined as three standard deviations above the mean OD of the negative control. According to the defined ODc, the strains were classified on the basis of their adherence ability into the following categories: nonadherent (OD ≤ ODc), weakly adherent (ODc < OD ≤ 2 × ODc), moderately adherent (2ODc < OD ≤ 4 × ODc), and strongly adherent (4 × ODc < OD) [40]. Results were expressed as means ± standard deviations (s.d.) of three independent experiments.

Statistical significance was obtained by using two-tailed, unpaired Student’s t-test with GraphPad Prism software (GraphPad Software Inc., La Jolla, CA, USA). Results were expressed as means ± standard deviations (s.d.) of three independent experiments. Differences were considered as statistically significant when *p*-values were <0.05 or <0.01.

OD_570_ values obtained by experiments conducted by using 96-well flat-bottomed plastic tissue culture plates was also defined as OD_570_/cm^2^, taking into consideration that the area of the bottom of the well is 0.33 cm^2^.

CFUs/mL counts arising from experiments carried out by using cut lengthwise 1cm segments of urinary catheters were represented as CFU/cm^2^, taking into consideration that the surface area of each segment is 1.65 cm^2^.

Recorded datasets obtained by CLSM were used for both visualization and semiquantitative analysis by counting pixels (2D) after thresholding the raw dataset.

### 4.13. Ethical Statement

”Biobank of the Fondazione Santa Lucia, Rome-Italy has been ethically approved as a research bank by the Ethical and Protocol Review Committee**,** with protocol identification number “CE/PROG.796”.

## 5. Conclusions

The P388-coated silicone urinary catheter shows promising potential as a safe and effective alternative to stem the relapse/recurrence of biofilm-related CAUTIs, providing more chances to treat these infections with antibiotics before the development of a mature and more resistant biofilm.

## Figures and Tables

**Table 1:** Percentages of area fractions covered by total biofilm and biofilm area constituted by dead cells as mean values of at least four images for each sample.

Treatment	Area Fraction Covered by Biofilm with Respect to 0.234 mm^2^	% of Biofilm Area Covered by Dead Cells
Unadsorbed channel inoculated with Ec5FSL	88.88%	7.87%
P388-adsorbed channel inoculated with Ec5FSL	0%	0%
Unadsorbed channel inoculated with Ec9FSL	92.00%	11.12%
P388-adsorbed channel inoculated with Ec9FSL	0%	0%
